# Towards Safer and More Sustainable Suspension PVC Manufacturing: A NuDIST-Based Inherent Safety Assessment

**DOI:** 10.3390/polym18202557

**Published:** 2026-10-17

**Authors:** Arelmys Bustamante-Miranda, Segundo Rojas-Flores, Ángel Darío González-Delgado

**Affiliations:** 1 Nanomaterials and Computer Aided Process Engineering Research Group (NIPAC), Chemical Engineering Department, Universidad de Cartagena, Cartagena 130015, Colombia; abustamantem@unicartagena.edu.co; 2 Institutos y Centros de Investigación, Universidad Cesar Vallejo, Trujillo 13001, Peru; srojasf@ucv.edu.pe

**Keywords:** polyvinyl chloride, NuDIST, inherent safety, wastewater regeneration, polymer manufacturing

## Abstract

Poly(vinyl chloride) (PVC) remains one of the most widely produced polymeric materials worldwide because of its extensive use in construction, packaging, medical devices, and other industrial applications. As PVC manufacturing is evolving toward more resource-efficient configurations, mass and energy integration and wastewater regeneration should be assessed together with process safety. This study applies the Numerical Descriptive Inherent Safety Technique (NuDIST), a continuous logistic function-based screening methodology, to an industrial-scale suspension PVC production process incorporating mass integration, energy integration, and wastewater regeneration under a zero-liquid-discharge (ZLD) approach. Chemical safety was evaluated through flammability, explosiveness, toxicity, and reactivity, whereas process safety considered temperature, pressure, heat of reaction, and the yield-based inventory proxy. The baseline assessment produced a Chemical Safety Total Score (CSTS) of 251.12 and a Process Safety Total Score (PSTS) of 21.20, giving an overall NuDIST score of 272.32. The chemical component accounts for approximately 92.2% of the total score. Under the adopted dataset, vinyl chloride monomer (VCM) supplies the maximum score for all four chemical parameters, while pressure is the largest process safety contribution. An illustrative one-at-a-time sensitivity analysis showed that a 10% reduction in maximum pressure decreases the total score to 270.21 and a 10% relative increase in yield decreases it to 268.35, whereas replacing the 1 ppm baseline VCM toxicity input with the 5 ppm 15 min regulatory short-term limit changes the total score by less than 0.01%. The results therefore identify VCM-related inherent hazards and process severity as the main priorities for subsequent inherently safer designs, while the NuDIST is interpreted as a screening tool rather than a probabilistic quantitative risk analysis.

## 1. Introduction

Plastics production has increased substantially over the last decades, due to the low cost, durability, flexibility, and versatility of these materials, while simultaneously increasing the challenges associated with plastics consumption and waste generation [1]. Gautam et al. described this expansion at the global level and highlighted the environmental and human health implications associated with increasing plastic production and disposal [2]. Rafey and Siddiqui emphasized the relevance of plastic waste management in sectors characterized by high material consumption, including packaging applications [3], whereas Eze et al. reviewed technological challenges associated with the treatment and valorization of plastic wastes [4]. Thompson et al. reviewed the evolution and current understanding of microplastic contamination [5], Gunawardhana et al. described the mechanisms through which plastics undergo environmental degradation and fragmentation [6], and Eriksen et al. estimated the presence of more than 170 trillion plastic particles afloat in the world’s oceans [7]. Within this broader context, Braun described the historical development of poly(vinyl chloride) (PVC) as a major industrial polymer [8], while Beltrán Domínguez documented its industrial production and the properties supporting its extensive use in construction and other applications [9]. A commercial technical source also summarizes common PVC product characteristics and industrial uses [10], whereas Kudzin et al. examined environmental concerns associated with PVC production, use, and end-of-life management [11]. At the manufacturing level, Prieto et al. identified wastewater treatment and water reuse as opportunities for improving resource management in PVC plants [12], Thornton discussed the environmental implications associated with chlorine-based PVC production [13], and Gao et al. demonstrated that suspension PVC polymerization requires careful control of reaction behavior, heat transfer, and monomer conversion [14].

Suspension PVC manufacturing involves safety challenges that must be considered within efforts to improve its environmental and resource performance. Ogle et al. analyzed an industrial explosion associated with flashing liquid in a process vessel, illustrating the importance of controlling pressure, temperature, and phase changes in process equipment [15]. In the specific context of PVC, Kudzin et al. reviewed environmental and health concerns associated with PVC and its production and disposal pathways, including hazards related to chlorine-containing substances and thermal degradation products [11]. Vinyl chloride monomer (VCM) is of particular concern because of its flammability, toxicity, and carcinogenicity; OSHA has established an 8 h permissible exposure limit of 1 ppm and a 15 min short-term limit of 5 ppm for vinyl chloride [16]. Richnák and Fidlerová examined the relevance of the Sustainable Development Goals to technological and industrial transformation [17], while Polo et al. reviewed advances in sustainable manufacturing aimed at improving resource and energy efficiency [18]. El-Halwagi established process integration as a systematic framework for resource conservation, pollution prevention, and recovery and reuse of material and energy streams [19], whereas Sonune and Ghate reviewed wastewater treatment technologies that support the treatment and potential reuse of industrial effluents [20]. These approaches provide a basis for reducing energy, material, and freshwater requirements in PVC manufacturing; however, recycle streams, heat recovery, and wastewater regeneration can also modify material circulation and operating conditions. Consequently, their implementation should be accompanied by an explicit assessment of process safety.

Risk assessment can be approached through methodologies that differ in scope, required information, and level of detail. General risk analysis frameworks include both quantitative and qualitative approaches for identifying hazards, estimating their significance, and supporting the selection of control measures [21]. In chemical process applications, Quantitative Risk Assessment (QRA) provides a structured means of estimating the probability and consequences of accident scenarios and is therefore particularly useful when detailed information is available [22]. Failure Mode and Effects Analysis (FMEA), together with its criticality-based extension by Srinivasan and Nhan, focuses on identifying potential failure modes and prioritizing them according to their effects and relative importance [23], whereas a Hazard and Operability Study (HAZOP) systematically examines deviations from intended operating conditions and their possible causes and consequences [24]. For earlier stages of process development, Heikkilä proposed the Inherent Safety Index (ISI), which combines chemical and process-related subindices to support the identification and comparison of inherent hazards before detailed protective systems are defined [25]. Ahmad et al. subsequently developed the Numerical Descriptive Inherent Safety Technique (NuDIST), in which flammability, explosiveness, toxicity, reactivity, temperature, pressure, heat of reaction, and process inventory are transformed into continuous numerical scores through logistic functions. Unlike discrete index-based scoring, this formulation reduces discontinuities between adjacent hazard ranges and provides a more gradual representation of parameter variation [26]. The NuDIST is therefore used in the present study as an early-stage inherent hazard screening methodology; however, it does not replace an HAZOP, FMEA/FMECA, or QRA, which can provide complementary information on operational deviations, failure modes, accident scenarios, and consequences.

Recent studies on suspension PVC production have provided complementary perspectives on resource efficiency and inherent safety. Guardo-Ruiz et al. evaluated a PVC production configuration incorporating mass and energy integration and direct water recycling through the Water–Energy–Product (WEP) methodology, identifying opportunities to improve the technical performance of the process from the perspective of water and energy use [27]. Mendivil-Arrieta et al. focused specifically on energy integration, applying pinch analysis to a large-scale PVC production process and subsequently evaluating its technical performance using the WEP framework [28]. From a safety perspective, González-Delgado et al. applied the Inherent Safety Index (ISI) to large-scale suspension PVC production to characterize the chemical and process hazards of the manufacturing route [29]. Bustamante-Miranda et al. subsequently evaluated an energy-integrated PVC production process incorporating water regeneration and analyzed the influence of this configuration on the chemical and process components of the ISI [30]. Because the ISI assigns hazard levels through predefined discrete scoring ranges, such an assessment provides an index-based characterization of the integrated system. In contrast, the continuous logistic functions of the NuDIST allow the numerical contributions associated with individual chemical properties and operating parameters to be represented without the abrupt transitions between adjacent discrete categories described by Ahmad et al. [26]. Outside PVC manufacturing, Zuorro et al. demonstrated the applicability of the NuDIST together with computer-aided process simulation for the inherent safety assessment of large-scale chitosan production, showing how process-derived information can be incorporated into the continuous NuDIST framework [31].

Accordingly, this study applies the Numerical Descriptive Inherent Safety Technique (NuDIST) to a suspension PVC production process incorporating mass integration, energy integration, and wastewater regeneration under a zero-liquid-discharge approach. The assessment quantifies the chemical safety contribution through flammability, explosiveness, toxicity, and reactivity, and the process safety contribution through the temperature, pressure, heat of reaction, and process inventory, following the continuous numerical formulation proposed by Ahmad et al. [26]. The objectives are to characterize the inherent hazard profile of the integrated PVC system, to identify the chemical properties and operating parameters that dominate the Chemical Safety Total Score (CSTS) and Process Safety Total Score (PSTS), and to establish quantitative priorities for subsequent safety improvement. The contribution of this work therefore lies in extending the safety characterization of the integrated PVC process through a NuDIST-based assessment rather than in modifying the original NuDIST formulation or proposing a new process configuration. In this way, the study provides a complementary inherent safety perspective for an integrated suspension PVC system that has not previously been characterized using this continuous numerical framework while recognizing that detailed accident scenarios and failure mechanisms require complementary risk analysis methodologies.

## 2. Materials and Methods

### 2.1. Process Description

The process data basis for the present assessment corresponds to the industrial-scale suspension PVC production model reported by Aguilar-Vásquez et al. [32]. That model integrates mass and energy recovery with wastewater regeneration and was developed using operating information from industrial PVC plants together with data reported in the specialized literature. The modeled plant has a PVC resin production capacity of 467,436.3 t/year, and the mass and energy balances are expressed using the production of 1 t of PVC as the calculation basis. The process configuration, stream conditions, and recycle structure reported in that model were adopted as the input dataset for the present inherent safety assessment. Accordingly, the purpose of this work is not to generate an independent process simulation, but to evaluate the inherent hazard characteristics of this defined industrial-scale configuration using the NuDIST. Figure 1 presents the process flow diagram and identifies the principal process, utility, recycle, and wastewater streams considered in the assessment.

PVC production begins with the combination of fresh and recovered vinyl chloride monomer (VCM), which are fed to the polymerization section at an 80/20 fresh-to-recycled ratio. This ratio corresponds to the operating configuration represented by Aguilar-Vásquez et al. [32] and reflects the recovery and internal reuse of unreacted VCM rather than its disposal as a residual stream. Polymerization takes place in an aqueous medium using poly(vinyl alcohol) (PVA) as stabilizer and (3-hydroxy-1,1-dimethylbutan-2-yl)-2-methylheptane as initiator. The reactor operates at approximately 70 °C and 9.81 bar, conditions that require continuous removal of heat because of the exothermic character of the polymerization. Bracho-Carmona and Van der Biest-Rodríguez specifically examined cooling water requirements in an industrial PVC polymerization area, illustrating the importance of adequate heat removal capacity during this stage [33]. Under the operating conditions and mass loading represented in the process model, VCM conversion is approximately 85% [32]. This conversion is therefore treated as a characteristic of the evaluated process configuration rather than as a universal conversion value for suspension PVC polymerization.

The reactor effluent consists of PVC particles suspended in water together with residual VCM and process additives. After leaving the polymerization section at approximately 70 °C and 3.43 bar, the stream is subjected to residual monomer recovery. The first separation occurs in a flash stage operated at approximately 1.77 bar, where nearly 95% of the free VCM is volatilized. The vapor stream also contains water and traces of PVA and initiator and is subsequently cooled, compressed, and condensed to recover VCM for internal recycling. Chan et al. described process modeling of PVC manufacture, including the principal polymerization, separation, and recovery operations, providing an additional process-level reference for this type of configuration [34]. The remaining PVC-rich suspension is subsequently treated by steam stripping to further reduce the residual monomer content. Steam is supplied at 13.73 bar and 225 °C, and the recovered VCM is condensed and returned to the process at approximately 3.43 bar and 8 °C.

The steam required by the stripping and associated thermal operations is supplied by a boiler with a nominal capacity of 20 t/h. This capacity corresponds to the steam requirement of the plant at the production scale and operating conditions represented in the process model; it is therefore part of the defined utility configuration rather than an independently optimized design variable in the present assessment. After residual VCM removal, thermal energy from the PVC suspension is recovered through heat exchange and used to preheat air for the drying section. The suspension, which contains approximately 70 wt% water, is then centrifuged at approximately 1800 rpm, removing about 75% of its water content. The resulting concentrated PVC paste contains approximately 25% moisture and is sent to the drying stage. Drying is performed using hot air supplied at approximately 250 °C while maintaining the PVC product temperature near 70 °C to limit thermal degradation [32]. Cyclones and bag filters are subsequently used for particle recovery, with the process configuration reporting approximately 99% particulate removal efficiency.

The aqueous streams generated principally during solid–liquid separation are directed to a wastewater regeneration train defined according to the characteristics of the process effluents and the mass integration strategy incorporated into the plant-wide configuration. Streams requiring treatment are first subjected to physicochemical operations comprising coagulation, flocculation, and clarification. The treated water is subsequently cooled to approximately 35 °C before entering anaerobic and aerobic biological stages aimed at degrading residual PVA and other biodegradable constituents. Reverse osmosis is applied as the final regeneration step, allowing treated water to be returned to the production system for process water requirements and steam generation. The treatment sequence therefore serves the water reuse structure of the integrated system and supports operation under the zero-liquid-discharge framework considered in this study.

For the NuDIST assessment, the system boundary encompasses the principal raw material, utility, product, wastewater, and recycle streams represented in Figure 1. The chemical species considered in the safety assessment were selected from substances explicitly present in these streams and from additives used in polymerization and water treatment. Table 1 lists the corresponding substances and CAS registry numbers, whereas Table 2 reports the temperature and pressure conditions of the streams used to determine the operating condition parameters of the NuDIST. Stream identifiers in Figure 1 correspond directly to those reported in Table 2, allowing the operating data used in the safety calculations to be traced throughout the process.

### 2.2. The Numerical Descriptive Inherent Safety Technique (NuDIST) for Inherent Safety Assessment in the PVC Production Process Integrated in Terms of Mass and Energy with Wastewater Regeneration

The Numerical Descriptive Inherent Safety Technique (NuDIST) was applied following the methodology proposed by Ahmad et al. [26] without modification of its original mathematical formulation. The NuDIST is an early-stage inherent safety assessment method that transforms chemical properties and process variables into continuous numerical hazard scores through parameter-specific logistic functions. The methodology was developed to reduce the discontinuities and subjective scaling associated with conventional index-based approaches, in which ranges of physical or chemical properties are assigned discrete hazard scores. In the NuDIST formulation, each evaluated parameter approaches a score of 0 as the corresponding inherent hazard decreases and a score of 100 as the hazard increases, allowing gradual differences among chemical properties and operating conditions to be represented numerically.

The assessment comprises two complementary components: Chemical safety is evaluated through flammability, explosiveness, toxicity, and reactivity, which are subsequently combined into the Chemical Safety Total Score (CSTS). Process safety is evaluated through the temperature, pressure, heat of reaction, and process inventory, whose contributions form the Process Safety Total Score (PSTS). The overall NuDIST score combines these two components and is interpreted as a relative indicator of inherent hazard, with lower scores representing lower inherent hazard levels when alternatives or processes are evaluated using the same methodology. Accordingly, the NuDIST is used in this study as an inherent hazard screening tool rather than as a probabilistic risk assessment or as a substitute for methodologies designed to evaluate specific accident scenarios, equipment failures, or operational deviations.

The operating information required for the process safety assessment was obtained from the industrial-scale suspension PVC process configuration reported by Aguilar-Vásquez et al. [32]. Chemical property data required for the chemical safety assessment—including the flash point, lower and upper explosive limits, occupational toxicity descriptors, and NFPA reactivity ratings—were compiled from the Safety Data Sheets and specialized chemical safety sources identified for each substance in the corresponding data tables. These property values were subsequently introduced into the original NuDIST logistic equations to determine the individual chemical and process safety parameter scores.

The use of physicochemical and operating data as the basis for early-stage safety screening is also supported by previous process safety studies. Hassim and Ali evaluated alternative methyl methacrylate production routes using inherent chemical process properties, demonstrating how property-based information can support the comparison of process alternatives before the detailed design is completed [55]. Green and Southard provide the standard chemical engineering framework for interpreting the thermophysical properties, operating conditions, and energy-related process variables that underlie quantitative process calculations and safety-related characterization [56]. From a complementary risk assessment perspective, Kim et al. examined a battery recycling process and applied risk estimation methods to identify and prioritize hazardous process conditions, illustrating the value of combining process information with systematic hazard assessment when evaluating industrial systems [57]. These contributions reinforce the use of structured process and property data for safety screening while also emphasizing that inherent hazard indicators such as the NuDIST should be interpreted within the specific scope of the methodology.

### 2.3. NuDIST Total Score

The overall NuDIST score combines the chemical and process safety contributions into a single inherent hazard indicator. Following Ahmad et al. [26], the NuDIST Total Score is calculated as the sum of the Chemical Safety Total Score (CSTS) and the Process Safety Total Score (PSTS), as expressed in Equation (1):

(1) NuDIST Total Score=CSTS+PSTS

A lower NuDIST Total Score represents a lower relative inherent hazard level when processes or alternatives are evaluated using the same methodological framework. The total score should therefore be interpreted together with its CSTS and PSTS components, because these values indicate whether the overall inherent hazard profile is governed primarily by chemical properties or by process operating conditions. The NuDIST Total Score is not a probability of accident occurrence, a consequence measure, or an absolute threshold separating safe and unsafe processes; rather, it is a comparative screening indicator based on the parameters incorporated into the methodology.

### 2.4. Chemical Safety Total Score (CSTS)

The Chemical Safety Total Score (CSTS) represents the chemical inherent hazard component of the NuDIST. Heikkilä [25] established the use of worst-case criteria in inherent safety screening [55], and Ahmad et al. [26] implemented this principle by evaluating four chemical parameters: flammability (SFL), explosiveness (SEXP), toxicity (STOX), and reactivity (SR). For each parameter, the maximum score obtained across the applicable substances is selected independently, and the four parameter-specific maxima are summed according to Equation (2):

(2) $$CSTS={\left(SFL\right)}_{max}+{\left(SEXP\right)}_{max}+{\left(STOX\right)}_{max}+{\left(SR\right)}_{max}$$

The four maxima are not required to originate from the same chemical substance [26]. Equation (2) therefore represents a process-level chemical inherent hazard envelope defined by the highest contribution of each chemical parameter; it is not obtained by first assigning a separate CSTS to every substance and then selecting the largest substance total.

Flammability (SFL) is evaluated from the flash point of each applicable substance. Ahmad et al. [26] use the flash point as the NuDIST descriptor because substances with lower flash points can generate ignitable vapor–air mixtures at lower temperatures and consequently receive higher scores. Equation (3) expresses the corresponding logistic function, where x is the flash point in degrees Celsius:

(3) $$S_{FL}=100\times (1-(\frac{1}{1+3.77e^{-0.024x}}))$$

Explosiveness (SEXP) is evaluated from the concentration interval over which a substance can form a combustible mixture with air. The lower explosive limit (LEL) represents the concentration below which the mixture is too lean to sustain combustion, whereas the upper explosive limit (UEL) represents the concentration above which the mixture is too rich. Ahmad et al. [26] therefore use UEL-LEL, expressed in vol%, as the input to Equation (4), where a wider range produces a higher explosiveness score:

(4) $$S_{EXP}=100\times \left(\left(\frac{1}{1+1096.63e^{-0.14x}}\right)\right)$$

Toxicity (STOX) is represented in the original NuDIST formulation by a short-term occupational exposure value in ppm, with lower allowable concentrations generating higher scores through Equation (5). For VCM, the baseline input used in this assessment is 1 ppm, corresponding to the OSHA 8 h permissible exposure limit. Because this value is a time-weighted average rather than a TLV-STEL, an additional sensitivity calculation was performed using OSHA’s 5 ppm limit averaged over a period not exceeding 15 min [56]. This treatment is reported explicitly so that the effect of the occupational exposure descriptor on the NuDIST score can be assessed rather than hidden within the baseline calculation.

(5) $$S_{TOX}=100\times \left(1-\left(\frac{1}{1+403.4288e^{-0.012x}}\right)\right)$$

The toxicity score should therefore be interpreted as an acute-exposure screening contribution, not as a complete toxicological characterization. Vinyl chloride is classified by the IARC as carcinogenic to humans (Group 1) [57], but IARC categories are qualitative and cannot be inserted directly into Equation (5). Likewise, LC50 values depend on the exposure route, duration, and units and are not dimensionally interchangeable with the ppm exposure input used to calibrate the NuDIST toxicity function. Incorporating those metrics numerically would require recalibration of the toxicity model rather than direct substitution.

Reactivity (SR) is evaluated using the NFPA instability/reactivity classification [58]. Ahmad et al. [26] employ the NFPA scale, which spans from 0 to 4, ranging from materials classified as stable to materials capable of detonation or explosive decomposition under specified conditions. Equation (6) converts the NFPA classification into the continuous NuDIST reactivity score:

(6) $$S_{R}=100\times \left(\frac{1}{1+270.43e^{-2.8x}}\right)$$

The NFPA reactivity levels considered in this study are summarized in Table 3. Equation (6) does not yield an exact score of zero for an NFPA classification of 0; substitution of x = 0 gives SR = 0.3684, rounded to 0.37. Accordingly, 0.37 is the minimum value mathematically generated by the NuDIST logistic function for the lowest NFPA reactivity classification and is not an independently assigned baseline.

The physicochemical and safety descriptors required for these calculations were compiled from the SDS/MSDS and specialized chemical safety sources identified for each substance. Table 4 and Table 5 report the adopted inputs, the resulting parameter scores, and the source or justification used for each value. When an applicable input was not available from the cited sources, the entry is reported as N/A rather than interpreted as evidence of zero hazard.

### 2.5. Process Safety Total Score (PSTS)

The Process Safety Total Score (PSTS) represents the process condition component of the NuDIST. Ahmad et al. [26] evaluate four parameters: temperature (ST), pressure (SP), heat of reaction (SHR), and process inventory (SPI). Following the worst-case structure of the method, the maximum score obtained for each parameter is retained and the four parameter-specific maxima are summed according to Equation (7). When several stages are present, the maxima may originate from different locations in the process.

(7) $$PSTS={\left(S_{T}\right)}_{max}+{\left(S_{P}\right)}_{max}+({S_{HR})}_{max}+({S_{PI})}_{max}$$

Temperature is considered because an elevated operating temperature increases the thermal severity of a process and the thermal stress imposed on equipment [25]. Ahmad et al. [26] developed separate logistic functions for temperatures above and below 25 degrees Celsius. Equations (8) and (9) are therefore applied according to the relevant temperature range, with x expressed in degrees Celsius. The maximum resulting ST value is retained for the PSTS calculation.

(8) $$S_{T>25{}^{\circ}C}=100\times \left(\frac{1}{1+403.43e^{-0.012x}}\right)$$

(9) $$S_{T<25{}^{\circ}C}=100\times \left(1-\left(\frac{1}{1+0.0025e^{-0.012x}}\right)\right)$$

Pressure is included because pressurized systems contain stored mechanical energy and impose greater demands on equipment integrity. Heikkilä [25] identifies pressure as a relevant inherent safety design variable, and Ahmad et al. [26] represent its contribution using Equation (10), where x is the operating pressure in bar. Higher pressures generate progressively higher SP values, and the maximum score identified in the process is retained for Equation (7):

(10) $$S_{PI}=100\times \left(\frac{1}{1+148.41e^{-0.2X}}\right)$$

The heat of reaction parameter represents the energetic contribution associated with chemical transformation. Felder and Rousseau [59] describe reaction enthalpy as the energy difference between products and reactants, while Ahmad et al. [26] distinguish endothermic and exothermic reactions through separate logistic functions. Equations (11) and (12) are applied according to the sign of the reaction enthalpy, with x expressed in kJ/mol. Larger departures from zero produce higher SHR contributions.

(11) $$S_{HR>0\;kJ/mol}=100\times \left(\frac{1}{1+601.85e^{-0.016x}}\right)$$

(12) $$S_{HR<0\;kJ/mol}=100\times \left(\frac{1}{1+403.43e^{0.006x}}\right)$$

The process inventory parameter requires specific interpretation. The NuDIST does not calculate the SPI from actual mass holdup, vessel volume, or plant inventory. Instead, Ahmad et al. [26] use percentage yield defined as the amount of product obtained under ideal conversion and selectivity conditions [59], as an early-design proxy for the amount of raw material required to produce the desired product. A higher yield implies that less raw material is required for a given product output and therefore produces a lower inventory score, whereas a lower yield produces a higher SPI. Equation (13) is thus interpreted in this study specifically as a yield-based inventory proxy rather than as a direct measurement of VCM or total plant holdup:

(13) $$S_{PI}=100\times \left(1-\frac{1}{1+1339.43e^{-0.12x}}\right)$$

## 3. Results and Discussion

### 3.1. Chemical Safety Total Score (CSTS) of the Suspension PVC Production Process

The chemical safety assessment considered VCM, water, oxygen, PVC, PVA, nitrogen, sodium hydroxide, sodium hypochlorite, citric acid, and potassium phosphate, as listed in Table 1. Table 4 and Table 5 report the adopted property inputs and the corresponding NuDIST scores. Entries for which an applicable property value was not available are reported as N/A and were not interpreted as evidence of zero hazard.

For flammability, VCM has a reported flash point of −78 °C and therefore produces the highest SFL value, 96.08, through Equation (3). PVA, with a flash point of 79 °C, produces a substantially lower score of 36.15. No applicable flash point value was used for the remaining substances in Table 4 and Table 5, so they do not participate in selection of the flammability maximum.

For explosiveness, VCM has a UEL-LEL interval of 29.40 vol%, which produces S_EXP_ = 5.30 through Equation (4). Citric acid has a much narrower reported interval of 2.01 vol% and produces S_EXP_ = 0.12. The other substances have no applicable explosive range input in the adopted dataset and therefore do not govern this parameter.

For toxicity, the baseline VCM input of 1 ppm produces S_TOX_ = 99.75 through Equation (5). This value is numerically close to the upper asymptote of the NuDIST toxicity function because the adopted exposure concentration is very low relative to the function midpoint. The 1 ppm value is the OSHA 8 h permissible exposure limit rather than a TLV-STEL [16], so its use is treated as a screening surrogate. PVA and PVC were not assigned a ppm short-term toxicity score because the cited sources do not provide an applicable input in the form required by Equation (5).

For reactivity, VCM has an NFPA classification of 2 and produces SR = 50.00, the maximum value among the evaluated substances. Sodium hydroxide and sodium hypochlorite, classified at level 1, produce SR = 5.73, whereas substances classified at level 0 produce SR = 0.37. The latter value is not an independently assigned baseline; Equation (6) gives 0.3684 at x = 0, which rounds to 0.37.

The underlying chemical property information was obtained from the SDS/MSDS and specialized sources identified in Table 4 and Table 5, including CAMEO Chemicals, ICSC records, manufacturer Safety Data Sheets, and occupational exposure sources. This source-by-source reporting makes the calculation traceable and also identifies where the NuDIST input is a screening surrogate rather than a directly reported TLV-STEL.

The process-level CSTS was calculated according to Equation (2) by summing the maximum score for each chemical parameter, not by calculating a separate CSTS for every substance. Under the baseline dataset, VCM happens to provide all four maxima: S_FL, max_ = 96.08, S_EXP, max_ = 5.30, S_TOX, max_ = 99.75, and S_R, max_ = 50.00. Their sum gives CSTS = 251.12, as summarized in Table 6. Thus, VCM is the governing chemical in the baseline NuDIST screening, but this result follows from parameter-wise maxima rather than from selecting the largest of several substance-level CSTS values. No separate CSTS is assigned to PVC, PVA, sodium hydroxide, or the other individual substances in the final process-level calculation.

The toxicity sensitivity case further shows that replacing the 1 ppm baseline VCM input with the OSHA 5 ppm 15 min short-term limit changes STOX from 99.75 to 99.74 and the CSTS from 251.12 to 251.11. The change is negligible because both values lie on the upper plateau of the logistic function. This numerical insensitivity should not be interpreted as evidence that chronic toxicity is unimportant; the IARC classifies vinyl chloride as carcinogenic to humans (Group 1) [57], a hazard dimension that Equation (5) does not encode.

Table 6 summarizes the parameter-wise maxima used to construct the CSTS. Because VCM supplies each maximum under the baseline inputs, the Figure 2 displays VCM for all four chemical safety contributions. The result identifies the dominant intrinsic chemical hazard profile but does not quantify exposure frequency, released mass, or the effectiveness of containment systems. The 1 ppm VCM toxicity input corresponds to the OSHA 8 h PEL and is used as a screening surrogate in the baseline NuDIST calculation; the 5 ppm 15 min OSHA limit is evaluated separately as a sensitivity case. The sodium hydroxide value is an occupational limit surrogate converted to ppm for screening and should not be interpreted as a formal TLV-STEL.

### 3.2. Process Safety Total Score (PSTS) of the PVC Production Process Integrated in Terms of Mass and Energy with Wastewater Regeneration

The process safety parameters are summarized in Table 7. The maximum temperature is 250 °C (stream 21 in Table 2), which gives ST = 4.74 through Equation (8). The maximum pressure is 13.73 bar (VAPOR stream in Table 2), which gives SP = 9.50 and is the largest baseline process safety contribution. The exothermic heat of reaction of −100 kJ/mol gives SHR = 0.45, while the 82.21% production yield gives SPI = 6.51 through the yield-based inventory proxy. These values are deterministic scores calculated from the adopted process inputs rather than statistical estimates.

The ranking of the baseline PSTS components is therefore pressure (9.50), then yield-based inventory proxy (6.51), then temperature (4.74), then heat of reaction (0.45). The pressure result identifies the most influential operating severity variable at the baseline point, whereas the SPI value should not be read as a direct measure of VCM holdup because Equation (13) uses yield rather than equipment inventory.

Using Equation (7), the four parameter-wise maxima give a PSTS of 21.20. Figure 3 illustrates the baseline contributions. To quantify how the mathematical NuDIST result responds to changes in its principal inputs, Table 8 presents an illustrative one-at-a-time sensitivity analysis in which each selected input is perturbed while the remaining baseline values are held constant. These cases are score sensitivity calculations and should not be interpreted as validated process redesigns or feasibility-tested optimization schemes.

The sensitivity results show that a 10% reduction in maximum pressure decreases SP from 9.50 to 7.39 and lowers the total NuDIST score from 272.32 to 270.21 (−0.78%). A 10% reduction in maximum temperature lowers the total to 271.14 (−0.44%). The largest modeled process-side reduction occurs for a 10% relative increase in yield, which decreases the SPI from 6.51 to 2.53, the PSTS to 17.22, and the total NuDIST score to 268.35 (−1.46%). By contrast, the alternative 5 ppm short-term VCM toxicity input changes the total by less than 0.01%. These results confirm that the overall ranking is dominated by the chemical safety component and that realistic reductions in operating severity would improve the score only modestly unless the dominant chemical hazard contribution is also addressed.

### 3.3. Total NuDIST Score

The baseline NuDIST Total Score was calculated using Equation (1). The CSTS of 251.12 and PSTS of 21.20 give an overall score of 272.32, as reported in Table 9 and Figure 4. Because the score is constructed from parameter-wise worst-case values, it should be interpreted as a relative inherent hazard screening indicator rather than as an accident probability or consequence metric.

The chemical safety component accounts for approximately 92.2% of the total NuDIST score, whereas the process safety component accounts for approximately 7.8%. The integrated PVC configuration is therefore governed primarily by the intrinsic hazard descriptors of the chemicals included in the NuDIST framework rather than by the baseline temperature, pressure, reaction enthalpy, and yield-based inventory scores.

### 3.4. Contextual Comparison with Previous NuDIST Applications

The NuDIST does not define universal numerical thresholds separating safe from unsafe processes; its total score is most defensible as a relative screening indicator within a clearly stated assessment scope [26]. Table 10 therefore provides contextual benchmarking against published NuDIST applications. The comparison is not intended as a like-for-like ranking of industrial sectors because the processes differ in chemistry, throughput, number of reaction steps, operating conditions, and the availability of inventory information.

Zuorro et al. applied the NuDIST to large-scale chitosan production from shrimp waste and reported CSTS = 171.01, PSTS = 209.20, and a total score of 380.20 [31]. In that system, ethanol strongly influences the flammability and explosiveness contribution, while the process safety component is much larger than in the present PVC case. This contrast illustrates how the balance between chemical and process contributions can change substantially even when the same NuDIST equations are used.

Ahmad et al. evaluated six methyl methacrylate (MMA) production routes with the original NuDIST formulation [26]. Their CSTS values range from 240 to 342 and their total scores from 332 to 574. The C2/PA route has the highest reported CSTS (342) and total score (574), whereas the TBA route has the lowest total score (332). The PVC CSTS of 251.12 lies within the MMA chemical safety range, but the PVC PSTS is substantially lower than the values reported for the MMA routes.

Additional full NuDIST applications broaden the context but also reinforce the need for caution. Moreno-Sader et al. reported CSTS = 185.88, PSTS = 371.35, and a total of 557.23 for an integrated shrimp-based biorefinery [60]. García-Maza et al. reported CSTS = 26.14, PSTS = 101.95, and a total of 128.09 for crude palm oil production [61]. These values reflect very different process chemistries and severity profiles and are used here only to illustrate the range of NuDIST responses across published applications.

Ahmad et al. also evaluated solvent alternatives for palm oil recovery using the chemical safety component of the NuDIST and reported CSTS values of 191.53 for cyclohexane and 198.32 for n-hexane [62]. Because that study did not report the PSTS or a complete NuDIST total for the solvent cases, those values are not included as full-process benchmarks in Table 10. They remain useful only as partial chemical safety references showing the influence of volatile organic solvents.

Table 10 therefore separates complete NuDIST assessments from partial chemical safety applications and reports only complete assessments in the numerical total score benchmark. Across the complete assessments, higher total scores can arise from severe chemical properties, demanding operating conditions, or both. However, the numerical differences should not be attributed to a single factor such as process scale because the NuDIST does not weight the maxima by throughput, equipment volume, or actual hazardous material holdup. Cross-process benchmarking is therefore descriptive rather than a direct measure of comparative industrial risk.

For the PVC process itself, the main value of the decomposition is the identification of intervention priorities. The baseline CSTS indicates that intrinsic VCM properties dominate the screening result, whereas the PSTS points first to pressure and then to the yield-based inventory proxy. These priorities can inform subsequent inherently safer design studies, but conventional safeguards such as containment, detection, and monitoring should be distinguished from changes that actually reduce inherent hazard.

### 3.5. Limitations of Cross-Process NuDIST Comparisons

The comparisons in Table 10 involve systems with different chemistries, production scales, numbers of processing stages, property datasets, and operating envelopes. Moreover, the NuDIST retains the maximum value of each parameter rather than weighting it by material flow or equipment inventory. A high-pressure condition in a small stream can therefore contribute the same SP as the same pressure in a high-throughput vessel. The reported scores are suitable for contextualizing the PVC result within published applications of the same methodology, but not for establishing a universal ranking of industrial risk.

The reported NuDIST scores are deterministic point estimates. Neither the original NuDIST formulation nor the comparison studies provide statistical input distributions from which confidence intervals could be derived. Accordingly, statistical confidence intervals are not reported here. Instead, Table 8 provides a transparent one-at-a-time sensitivity calculation for selected PVC inputs; a full probabilistic uncertainty analysis would require documented distributions or uncertainty bounds for the underlying chemical property and process model data.

## 4. Limitations of the NuDIST Methodology

The NuDIST provides a continuous numerical screening framework, but the resulting profile remains dependent on the selected descriptors and on the worst-case maximum used for each parameter. The CSTS and PSTS are not weighted by mass flow rate, exposure duration, equipment volume, or actual material holdup. In addition, the SPI is represented by production yield rather than the physical inventory. Consequently, the NuDIST score should not be interpreted as proportional to the actual quantity of hazardous material or energy present at a particular location in the plant.

The chemical property inputs also impose limitations. NFPA reactivity is categorical even though it is transformed through a continuous logistic equation, and a classification of 0 produces the mathematical minimum SR = 0.37 rather than zero. The toxicity function was developed for short-term occupational exposure and does not independently represent chronic toxicity or carcinogenicity. This is particularly relevant for VCM, which is classified by the IARC as carcinogenic to humans (Group 1) [57]. The heat of reaction term similarly condenses thermal behavior into a single enthalpy value and does not represent reaction kinetics, heat removal failure, runaway dynamics, or relief system performance.

Several PVC-specific hazard scenarios also lie outside the NuDIST parameter set. Fine PVC particles handled during drying, cyclone separation, and bag filtration may form combustible dust clouds and therefore require dedicated characterization of dust explosibility, ignition sensitivity, electrostatic charging, and explosion propagation. Kletz and Amyotte discuss dust explosion prevention and mitigation as a distinct, inherently safer design problem requiring specific analysis beyond conventional process hazard descriptors [63]. Kudzin et al. further reviewed the environmental and health hazards associated with PVC and its thermal degradation pathways [11]. More specifically, Zhang et al. examined the formation of polychlorinated dibenzo-p-dioxins and dibenzofurans (PCDD/Fs) during PVC combustion and fires and showed that their formation depends strongly on combustion conditions and material formulation [64]. These combustible dust and chlorinated thermal decomposition hazards are not explicitly represented by the four NuDIST chemical descriptors or by its temperature and heat of reaction scores. Consequently, the NuDIST result should not be interpreted as a complete characterization of these PVC-specific scenarios, which require dedicated dust explosion testing, fire and thermal decomposition analysis, and scenario-based safety assessment.

Accordingly, the present results should be used as an initial inherent hazard screen. An HAZOP can examine process deviations and operating mistakes in PVC systems [24]; FMEA/FMECA can address equipment and functional failure modes; and QRA can estimate scenario frequencies and consequences [22]. For the solids handling section, dedicated dust explosion testing and electrostatic ignition assessment would strengthen the evaluation, while thermal decomposition studies would be required for chlorinated degradation scenarios. Future work should also combine process model sensitivity runs with documented uncertainty ranges and, where possible, actual equipment inventories.

## 5. Conclusions

This study applied the NuDIST to an industrial-scale suspension PVC production process incorporating mass integration, energy integration, and wastewater regeneration under a zero-liquid-discharge (ZLD) approach. The assessment is a deterministic inherent hazard screen based on the original NuDIST equations and on the defined process dataset; it is not a probabilistic QRA or a claim of absolute process safety.

The baseline NuDIST score is 272.32, comprising CSTS = 251.12 and PSTS = 21.20. Chemical safety therefore contributes approximately 92.2% of the total. Under the adopted dataset, VCM provides the maximum flammability, explosiveness, toxicity, and reactivity scores and consequently governs the process-level CSTS. This result identifies VCM as the principal intrinsic chemical hazard priority, but conventional containment, monitoring, and detection systems would reduce exposure or consequence rather than the property-based CSTS itself. Within inherently safer design, the most direct chemical-side opportunity is therefore minimization of VCM holdup where technically feasible; substitution is intrinsically constrained because VCM is the monomer required for PVC production.

Process safety contributes 7.8% of the baseline total, with pressure (9.50) followed by the yield-based inventory proxy (6.51), temperature (4.74), and heat of reaction (0.45). The one-at-a-time sensitivity calculations quantify the direction of these priorities: a 10% reduction in maximum pressure lowers the total NuDIST score to 270.21, a 10% reduction in maximum temperature lowers it to 271.14, and a 10% relative increase in yield lowers it to 268.35. These values are mathematical sensitivity cases rather than feasibility-tested process modifications, but they show that moderation of pressure and temperature and improvements that reduce the yield-based inventory proxy can decrease the PSTS while leaving the dominant CSTS essentially unchanged.

Framed using the inherently safer design principles described by Kletz and Amyotte [63], the results support minimization of hazardous VCM inventory, moderation of operating severity, and simplification of handling and recycle arrangements as the most relevant directions for subsequent design studies. Protective measures such as leak detection, containment, ventilation, and emergency response remain necessary but should be recognized as safeguards rather than reductions in inherent hazard. A complete safety case for PVC manufacture should therefore combine the NuDIST with an HAZOP/FMEA and QRA, together with dedicated assessment of combustible dust and chlorinated thermal decomposition scenarios. The present work provides a quantitative baseline and prioritization framework for those subsequent analyses.

## Figures and Tables

**Figure 1:**
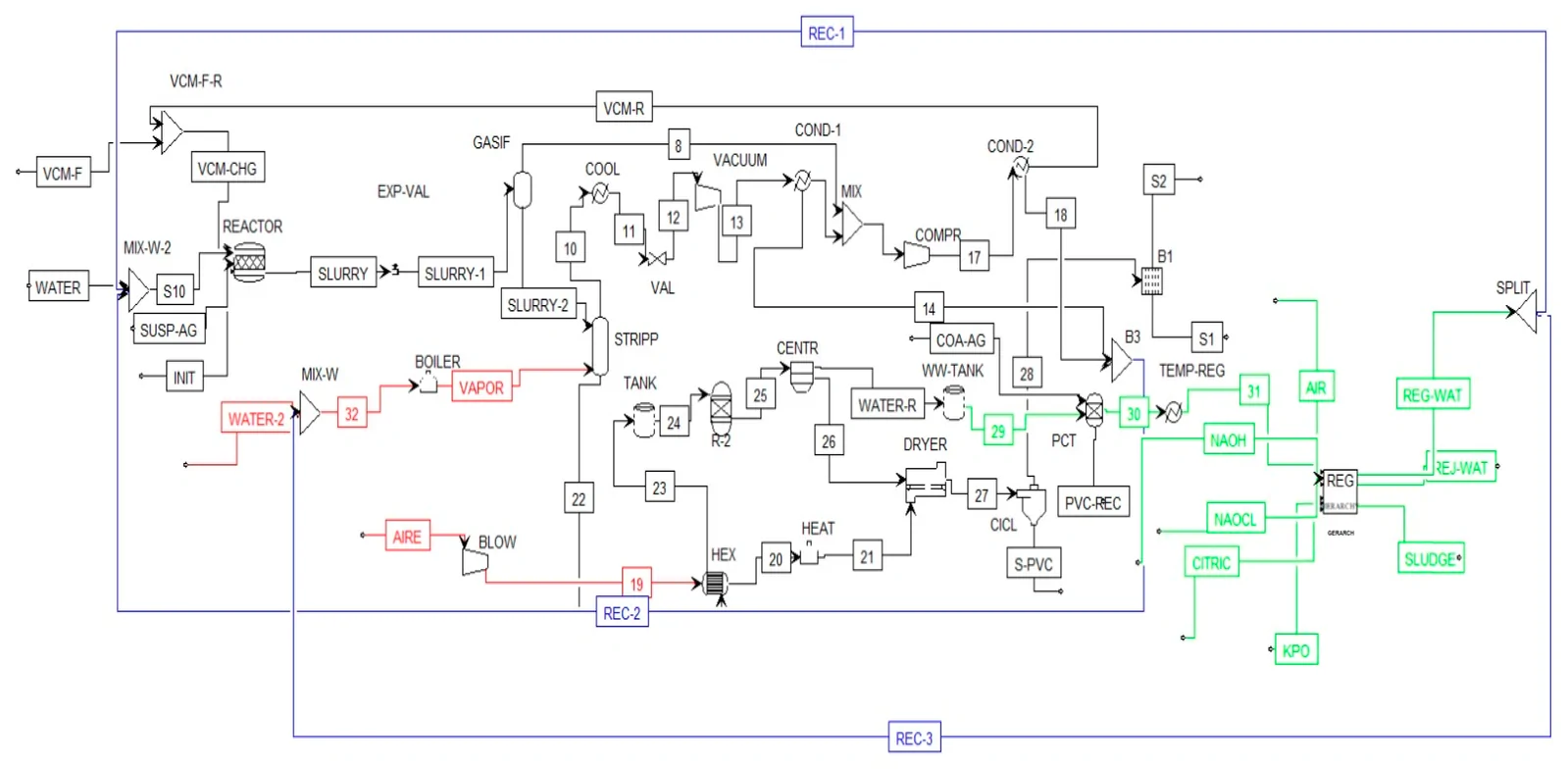
Flow diagram of the PVC suspension process with wastewater regeneration.

**Figure 2:**
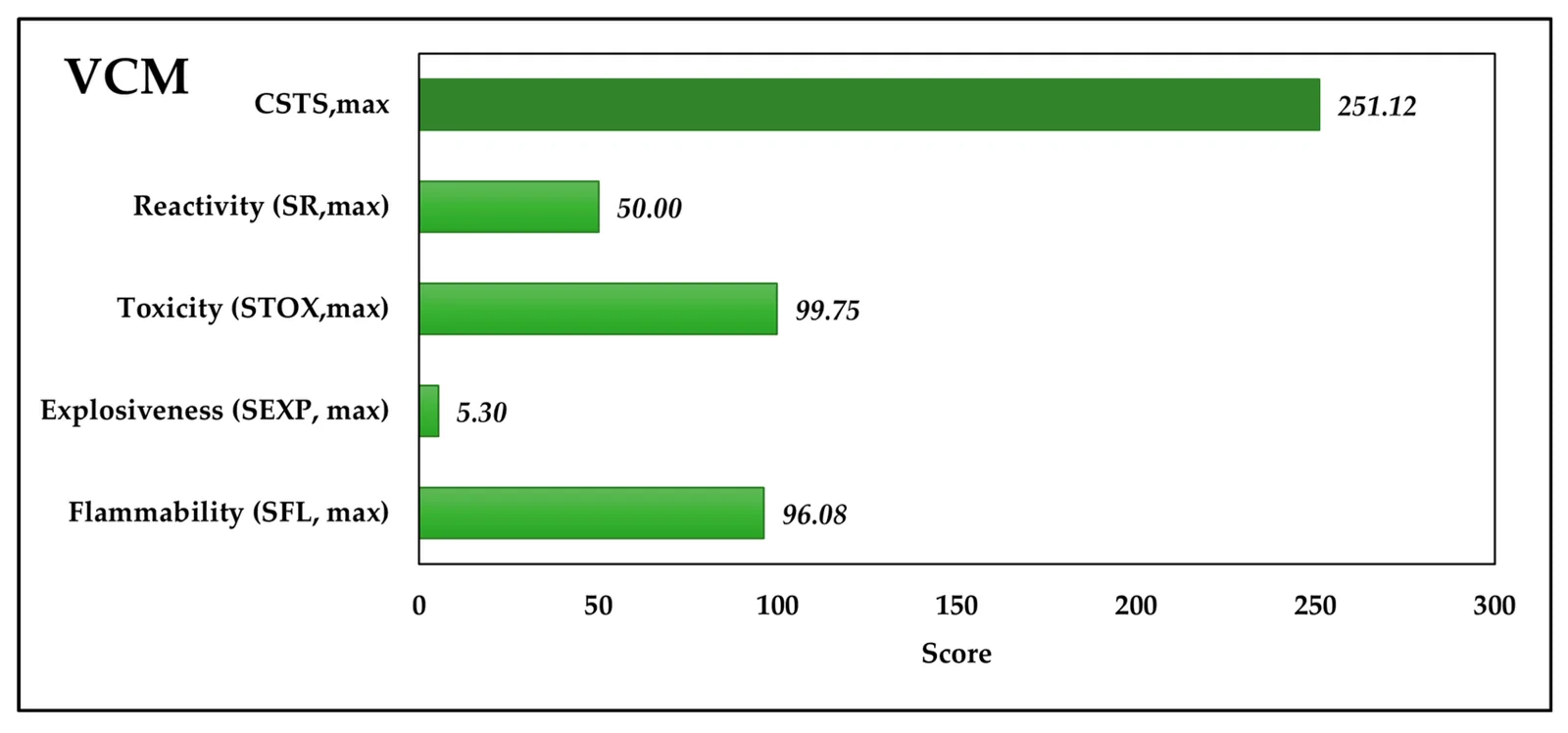
Chemical safety parameter maxima and process-level Chemical Safety Total Score (CSTS) for the integrated suspension PVC production process.

**Figure 3:**
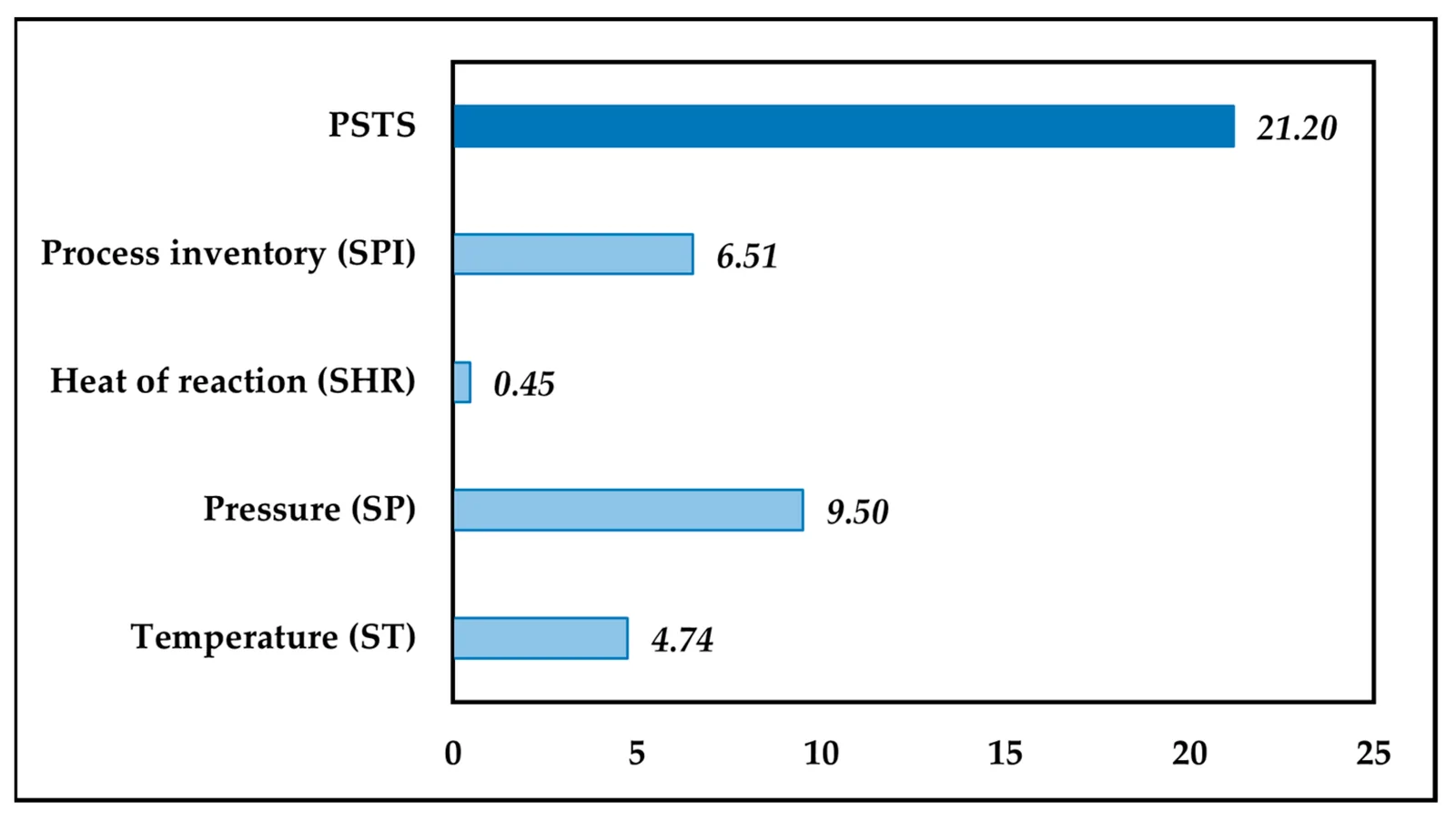
Baseline process safety parameter contributions and Process Safety Total Score (PSTS) for the integrated suspension PVC production process.

**Figure 4:**
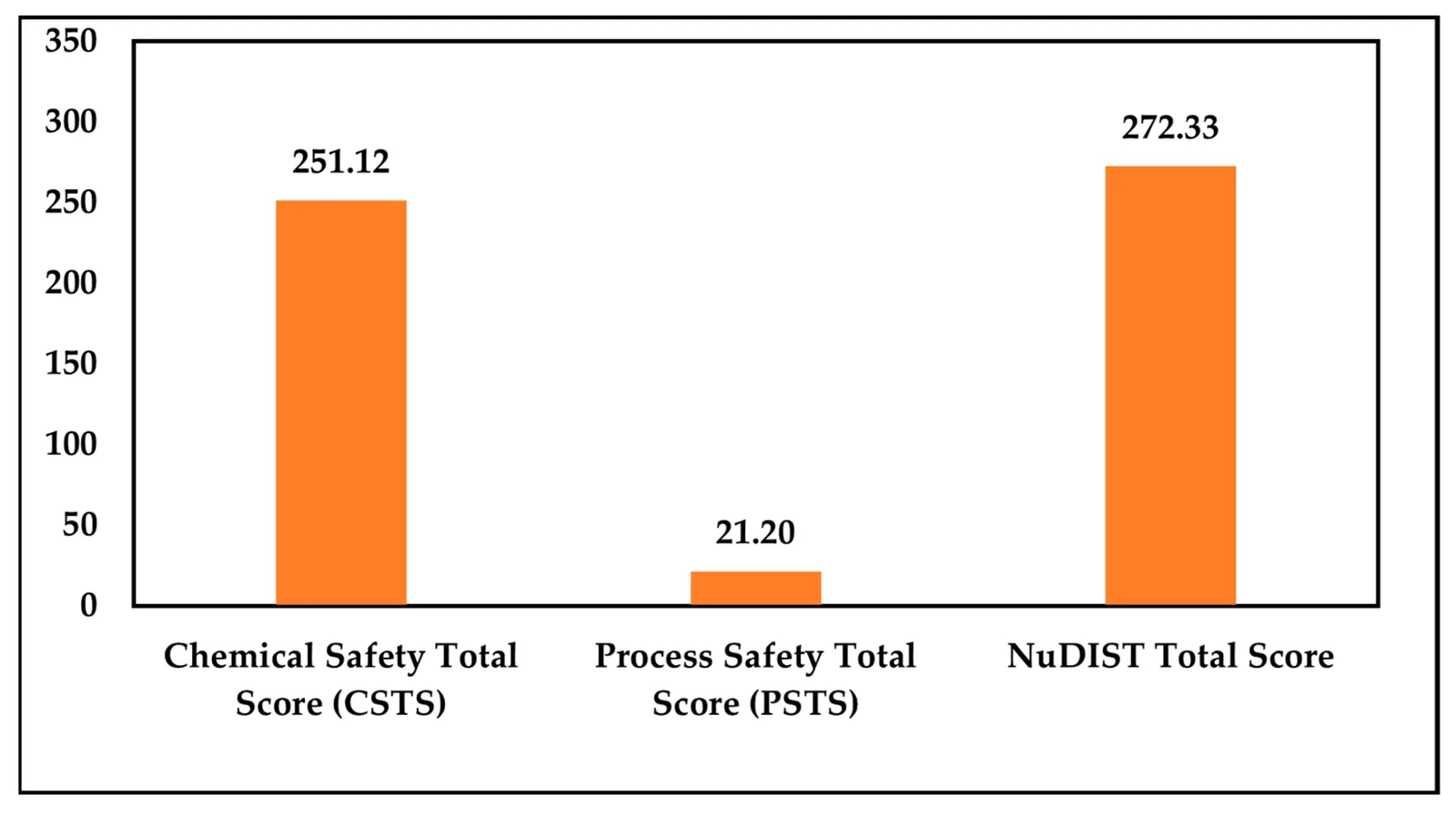
Baseline Chemical Safety Total Score (CSTS), Process Safety Total Score (PSTS), and NuDIST Total Score for the integrated suspension PVC production process.

**Table 1:** Main components of the mass- and energy-integrated suspension PVC production process with wastewater regeneration.

Main Components	CAS Number	Reference
Vinyl chloride monomer	75-01-4	[35,36,37]
Water	7732-18-5	[38]
Oxygen	7782-44-7	[39,40]
Poly(vinyl chloride)	9002-86-2	[41]
Poly(vinyl alcohol)	9002-89-5	[42,43]
Nitrogen	7727-37-9	[44,45]
Sodium hydroxide	1310-73-2	[46,47,48,49]
Sodium hypochlorite	7681-52-9	[50,51]
Citric acid	77-92-9	[52,53]
Potassium phosphate, monobasic	7778-77-0	[54]

**Table 2:** Operating conditions of the streams in the PVC production process integrated in terms of mass and energy with wastewater regeneration.

Stream Name	Temperature	Pressure
Units	°C	Bar
8	70.00	1.77
10	116.34	1.77
11	50.00	1.01
12	47.96	0.15
13	50.37	2.45
14	50.00	2.45
15	50.00	2.45
16	68.69	1.01
17	179.67	4.41
18	8.00	4.41
19	46.26	1.13
20	91.00	1.13
21	250.00	1.01
22	107.05	1.77
23	73.72	1.77
24	65.00	1.01
25	60.00	1.01
26	65.00	1.01
27	70.00	1.01
28	70.00	1.01
29	65.00	1.01
30	64.24	1.00
31	25.00	1.01
32	28.85	1.00
AIR	25.00	1.00
AIRE	32.00	1.01
CITRIC	25.00	1.00
COA-AG	25.00	1.00
INIT	32.00	10.00
KPO	25.00	1.00
NAOCL	25.00	1.00
NAOH	25.00	1.00
PVC-REC	64.24	1.00
REC-1	25.00	1.00
REC-2	47.95	1.01
REC-3	25.00	1.00
REG-WAT	25.00	1.00
REJ-WAT	25.00	1.00
S1	70.00	0.99
S2	70.00	0.99
S10	51.43	3.50
S-PVC	70.00	1.01
SLUDGE	25.00	1.00
SLURRY	70.00	10.00
SLURRY-1	66.04	1.78
SLURRY-2	70.00	1.77
SUSP-AG	32.00	10.00
VAPOR	225.00	13.73
VCM-CHG	32.26	4.90
VCM-F	32.00	4.41
VCM-R	8.00	4.41
WATER	85.00	3.43
WATER-2	32.00	0.99
WATER-R	65.00	1.01

**Table 3:** Reactivity levels according to the NFPA classification.

Score	Classification	Description
0	Stable	The substance remains unchanged and does not react upon contact with water.
1	Caution	The substance may undergo some reaction when exposed to heat or water, without exhibiting violent behavior.
2	Warning	The substance exhibits instability or may react violently in the presence of water.
3	Danger	The substance may exhibit explosive behavior under conditions such as impact, high temperatures, or confinement.
4	Extreme Danger	The material is capable of detonating or reacting in an extremely violent manner even under normal conditions.

**Table 4:** Chemical property inputs and NuDIST parameter scores for VCM, water, oxygen, PVA, and nitrogen.

Chemical Substance	Flash Point/SFL	UEL-LEL/SEXP	Toxicity Input/STOX	NFPA/SR	Source/Justification
VCM	−78 °C/96.08	29.40 vol%/5.30	1.00 ppm */99.75	2/50.00	PT Sulfindo [35]; NOAA [36]; ILO [37,38]; OSHA [16]. Scores from Equations (3)–(6).
Water	N/A	N/A	N/A	0/0.37	Process component; no applicable flash point, explosive range, or ppm toxicity input in the adopted dataset. SR from Equation (6).
Oxygen	N/A	N/A	N/A	0/0.37	NOAA [39]; ILO [40]. SR from Equation (6).
Poly(vinyl alcohol) (PVA)	79 °C/36.15	N/A	N/A	N/A	ILO [42] reports flash point and dust explosion potential; NOAA [43]. SFL from Equation (3).
Nitrogen	N/A	N/A	N/A	0/0.37	ILO [44]; NOAA [45]. SR from Equation (6).

* The 1.00 ppm toxicity input for VCM corresponds to the OSHA 8 h permissible exposure limit (PEL) and is used as an acute-exposure screening surrogate in the baseline NuDIST calculation, not as a probabilistic exposure estimate.

**Table 5:** Chemical property inputs and NuDIST parameter scores for sodium hydroxide, sodium hypochlorite, citric acid, potassium phosphate, and PVC.

Chemical Substance	Flash Point/SFL	UEL-LEL/SEXP	Toxicity Input/STOX	NFPA/SR	Source/Justification
Sodium hydroxide	N/A	N/A	1.22 ppm †/99.75	1/5.73	Tricon [46]; Carl Roth [47]; ILO [48]; OSHA [49]. Toxicity input is an occupational limit surrogate; scores from Equations (5) and (6).
Sodium hypochlorite	N/A	N/A	N/A	1/5.73	Sterilex [50]; Carl Roth [51]. SR from Equation (6).
Citric acid	N/A	2.01 vol%/0.12	N/A	0/0.37	NOAA [52]; Sucroal [53]. SEXP and SR from Equations (4) and (6).
Potassium phosphate, monobasic	N/A	N/A	N/A	0/0.37	Universidad Veracruzana SDS [54]. SR from Equation (6).
Poly(vinyl chloride) (PVC)	N/A	N/A	N/A	0/0.37	Aldrich SDS [41]. No ppm short-term toxicity input was used for PVC; SR from Equation (6).

† The 1.22 ppm toxicity input for sodium hydroxide is an occupational exposure limit surrogate converted to ppm for screening purposes and should not be interpreted as a formal TLV-STEL.

**Table 6:** Parameter-wise maxima used to calculate the process-level Chemical Safety Total Score (CSTS).

CSTS Parameter	Maximum NuDIST Score	Governing Substance	Source/Justification
Flammability (SFL, max)	96.08	VCM	Table 4; Equation (3)
Explosiveness (SEXP, max)	5.30	VCM	Table 4; Equation (4)
Toxicity (STOX, max)	99.75	VCM	Table 4; Equation (5)
Reactivity (SR, max)	50.00	VCM	Table 4; Equation (6)
Chemical Safety Total Score (CSTS)	251.12	-	Sum of parameter-wise maxima according to Equation (2)

**Table 7:** Baseline process safety inputs, NuDIST scores, and source/justification for the integrated suspension PVC process.

Parameter	Unit	Process Value	NuDIST Score	Source/Justification
Maximum temperature (ST)	°C	250.00	4.74	Table 2, stream 21; process configuration [32]; Equation (8)
Maximum pressure (SP)	bar	13.73	9.50	Table 2, VAPOR stream; process configuration [32]; Equation (10)
Heat of reaction (SHR)	kJ/mol	−100.00	0.45	Reaction data adopted for the process [32]; Equation (12)
Production yield/inventory proxy (SPI)	%	82.21	6.51	Process production data [32]; yield interpretation from Ahmad et al. [26]; Equation (13)
Process Safety Total Score (PSTS)	-	-	21.20	Sum of parameter-wise maxima according to Equation (7)

**Table 8:** Illustrative one-at-a-time sensitivity of selected NuDIST inputs for the integrated suspension PVC process. All non-varied baseline inputs are held constant; the scenarios quantify score sensitivity and do not represent validated process redesigns.

Illustrative Sensitivity Case	Changed Input	Affected Parameter Score	PSTS	NuDIST Total	Change in Total
Baseline	-	-	21.20	272.32	-
VCM short-term toxicity case	1 ppm → 5 ppm	STOX: 99.75 → 99.74	21.20	272.31	<−0.01%
10% lower maximum pressure	13.73 → 12.36 bar	SP: 9.50 → 7.39	19.09	270.21	−0.78%
10% lower maximum temperature	250 → 225 °C	ST: 4.74 → 3.56	20.01	271.14	−0.44%
10% relative increase in yield	82.21 → 90.43%	SPI: 6.51 → 2.53	17.22	268.35	−1.46%

**Table 9:** CSTS, PSTS, and NuDIST Total Score for the suspension PVC production process integrated in terms of mass and energy with wastewater regeneration.

Chemical Safety Total Score (CSTS)	Process Safety Total Score (PSTS)	NuDIST Total Score
251.12	21.20	272.32

**Table 10:** Contextual comparison of complete NuDIST assessments reported for the integrated suspension PVC process and selected published applications.

Process/Route	CSTS	PSTS	NuDIST Total	Assessment Scope	Reference
Suspension PVC process	251.12	21.20	272.32	Complete NuDIST assessment	This work
Chitosan production	171.01	209.20	380.20	Complete NuDIST assessment	[31]
Shrimp-based biorefinery	185.88	371.35	557.23	Complete NuDIST assessment	[60]
Crude palm oil production	26.14	101.95	128.09	Complete NuDIST assessment	[61]
MMA: ACH route	258	241	499	Complete NuDIST route assessment	[26]
MMA: C2/PA route	342	232	574	Complete NuDIST route assessment	[26]
MMA: C2/MP route	330	163	493	Complete NuDIST route assessment	[26]
MMA: C3 route	332	199	531	Complete NuDIST route assessment	[26]
MMA: i-C4 route	240	131	371	Complete NuDIST route assessment	[26]
MMA: TBA route	240	92	332	Complete NuDIST route assessment	[26]

## Data Availability

The data supporting the findings of this study are available from the corresponding author upon reasonable request.

## Abbreviations

The following abbreviations are used in this manuscript:

Abbreviation	Definition
PVC	Poly(vinyl chloride)
VCM	Vinyl chloride monomer
PVA	Poly(vinyl alcohol)
NuDIST	Numerical Descriptive Inherent Safety Technique
CSTS	Chemical Safety Total Score
PSTS	Process Safety Total Score
ZLD	Zero liquid discharge
WEP	Water–Energy–Product
NFPA	National Fire Protection Association
SDS/MSDS	Safety Data Sheet/Material Safety Data Sheet
ICSC	International Chemical Safety Card
LEL	Lower explosive limit
UEL	Upper explosive limit
QRA	Quantitative Risk Assessment
FMEA/FMECA	Failure Mode and Effects Analysis/Failure Mode, Effects and Criticality Analysis
HAZOP	Hazard and Operability Study
